# Supplementary material for: Retinal organoids derived from rhesus macaque iPSCs undergo accelerated differentiation compared to human stem cells
Source: Cell Prolif. 2022 Feb 15;55(4):e13198. doi: 10.1111/cpr.13198 (PMC9055909; doi:10.1111/cpr.13198)

S1 hPSC and rhiPSC characterization

1. Location of Characterization Data

| Cell Line | Data | Location |
| --- | --- | --- |
| rhiPSC89 | G banding, pluripotency immunocytochemistry, reprogramming construct clearance, and teratoma formation. | <https://doi.org/10.1016/j.scr.2017.03.011>  See Figure 1 |
| rhiPSC90 | G banding, pluripotency immunocytochemistry, reprogramming construct clearance, and teratoma formation. | <https://doi.org/10.1016/j.scr.2016.09.015>  See Figure 1 |
| rhiPSc2431 | G banding, pluripotency immunocytochemistry, reprogramming construct clearance, and alkaline phosphatase assay. | For complete methodology of these assays please see previously published. <https://doi.org/10.1016/j.stemcr.2015.12.010>  G-banding: Figure B in this supplementary.  Pluripotency immunocytochemistry: Figure C in this supplementary  Reprogramming construct clearance: Figure D in this supplementary.  Alkaline phosphatase assay: Figure E in this supplementary. |
| H9 | G banding and identity STR. | <https://www.wicell.org/product-files/productInfo/tr_RB66492.pdf>  Product Information & Testing datasheet for our lot number of cells we obtained directly from WiCell. |

1. G-Banding Analysis of rhIPSC2431 line.


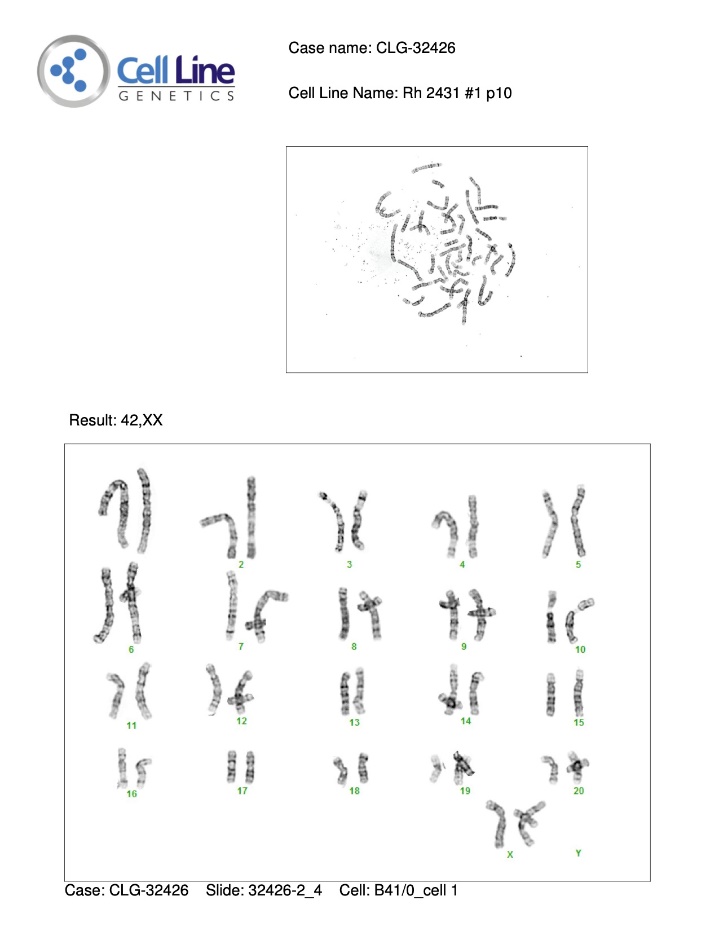

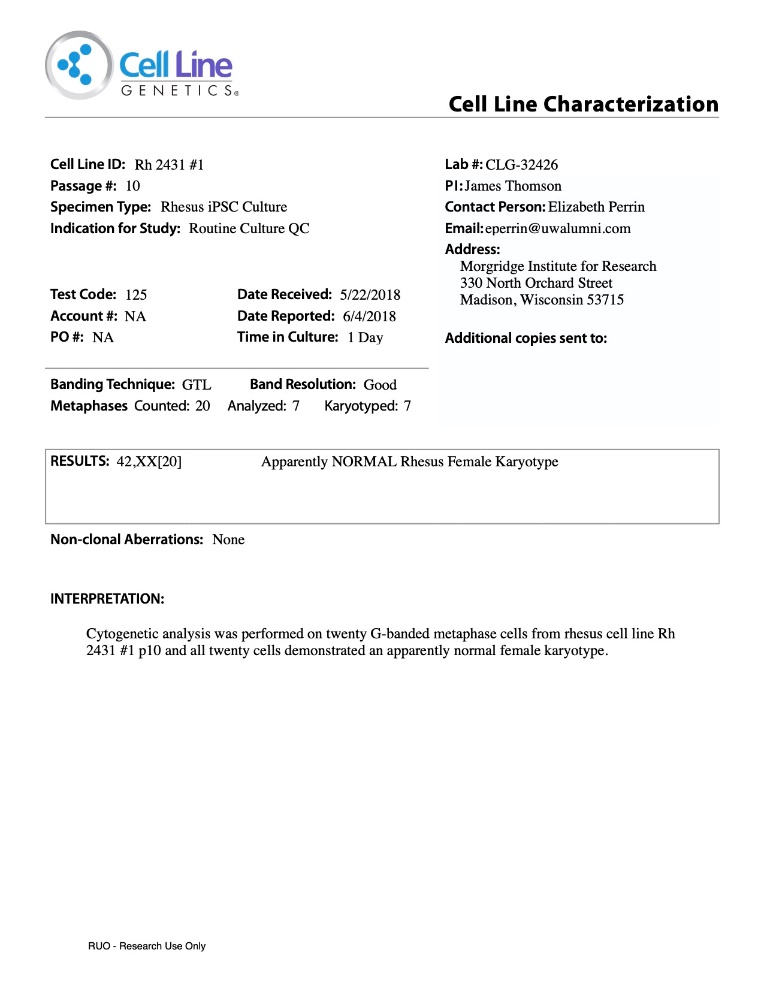


1. Pluripotency immunocytochemistry of rhIPSC2431 line

Nanog

Oct4


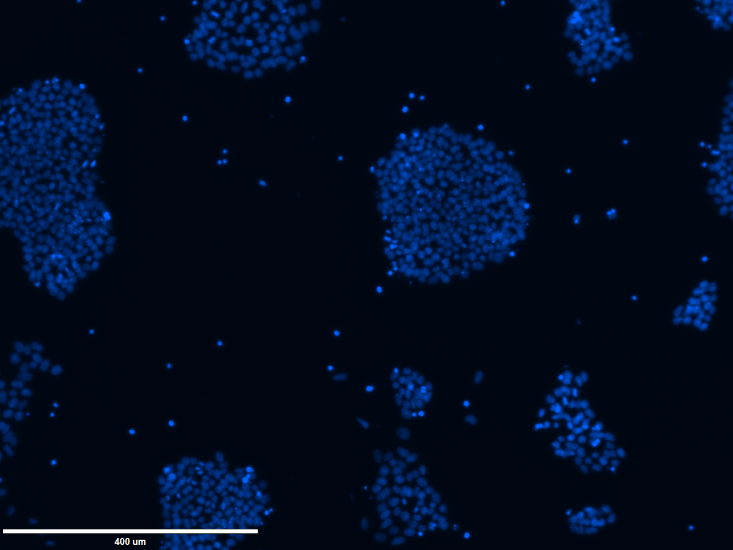

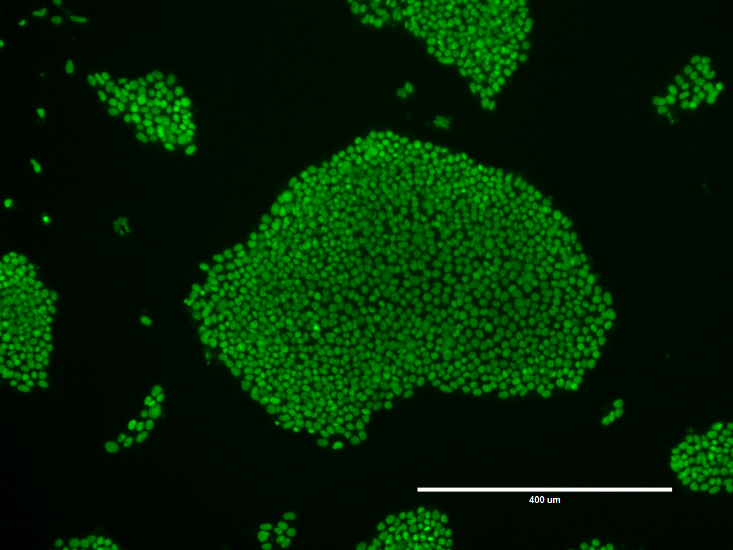

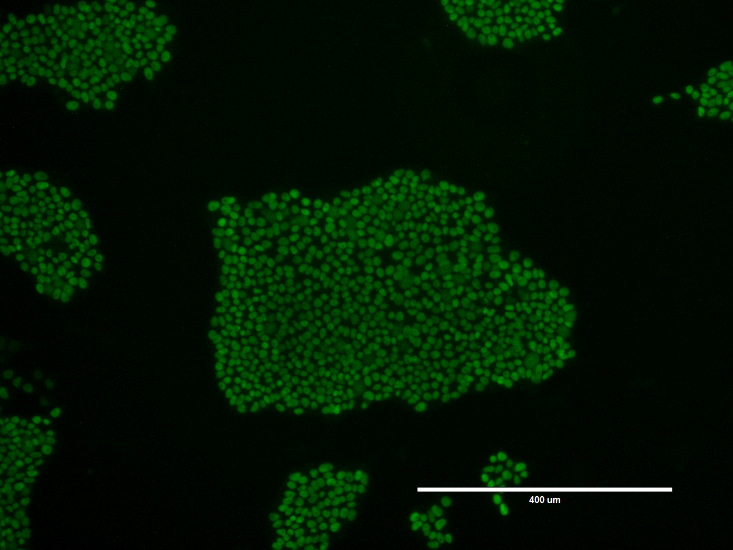

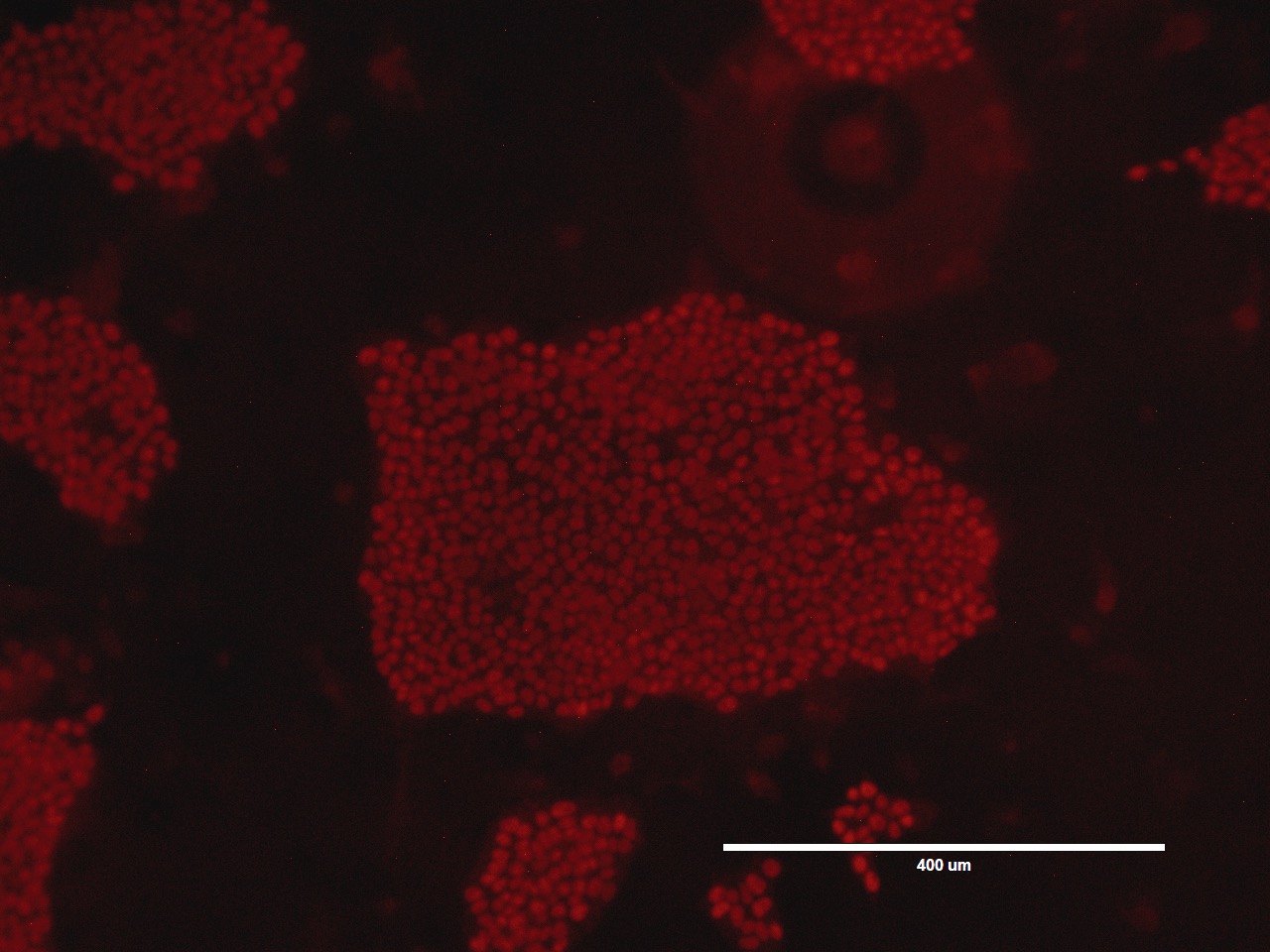


DAPI

Sox2

1. Reprogramming construct clearance of rhIPSC2431 line


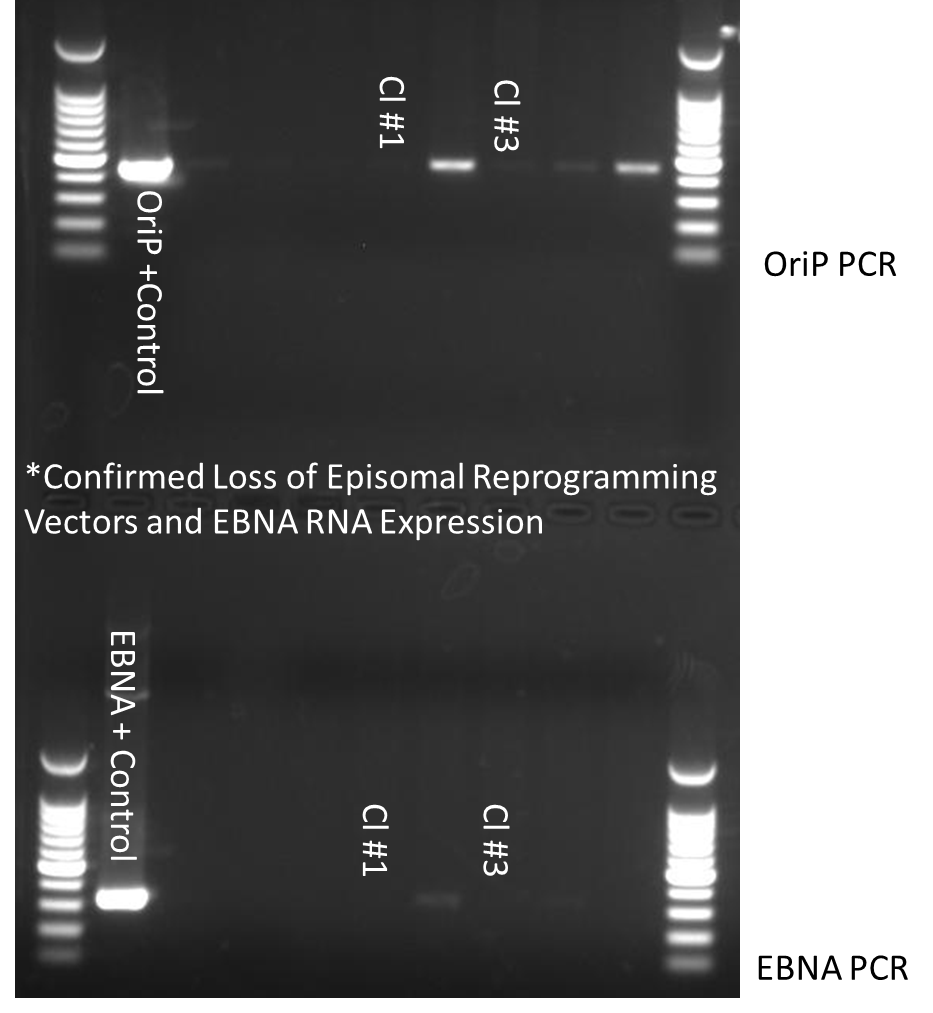


1. Alkaline phosphatase assay of rhIPSC2431 line


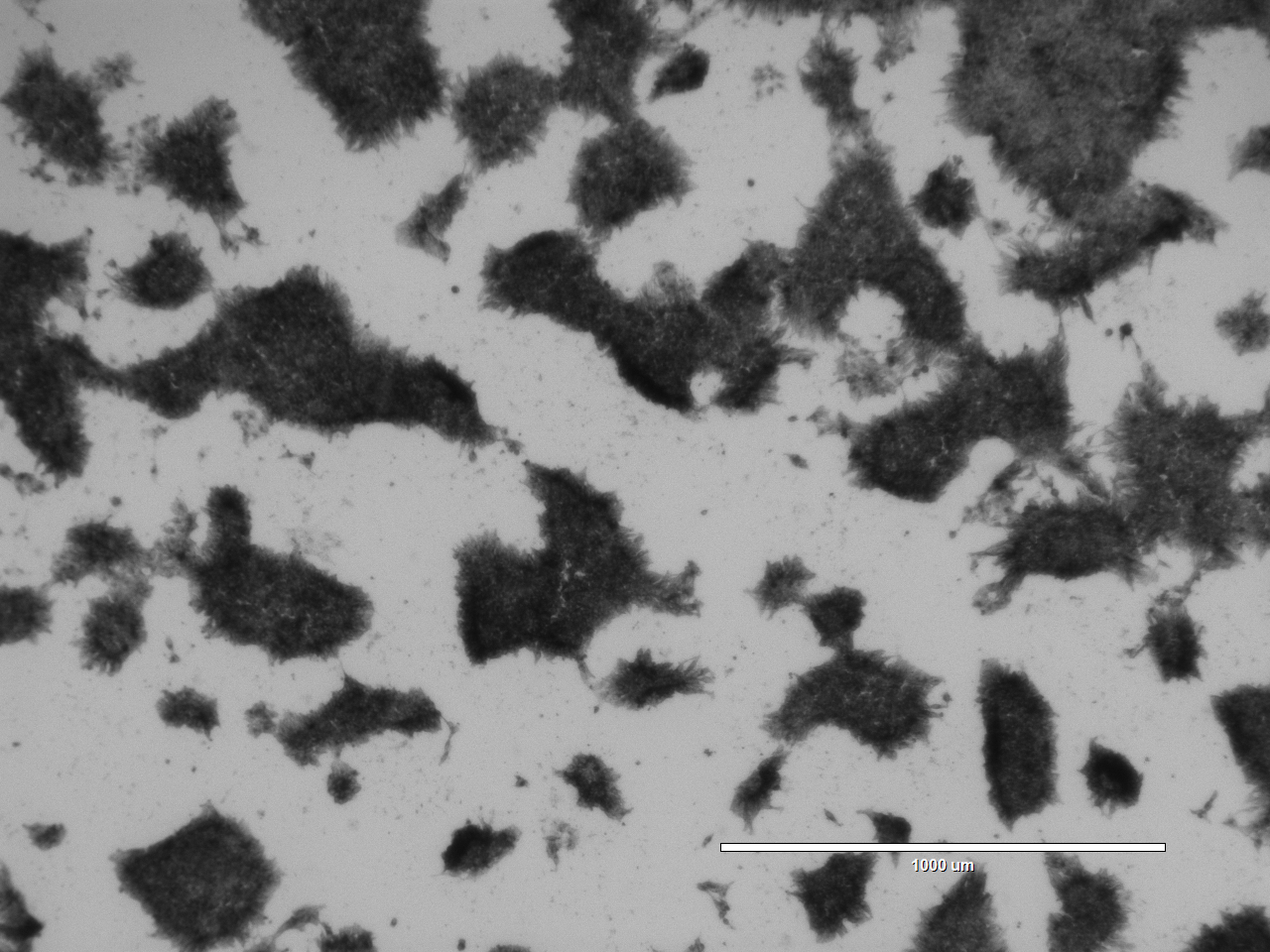

Supplement: Supplementary file 1 — Figure S1 [file CPR-55-e13198-s007.docx]
