# Supplementary material for: Retinal organoids derived from rhesus macaque iPSCs undergo accelerated differentiation compared to human stem cells
Source: Cell Prolif. 2022 Feb 15;55(4):e13198. doi: 10.1111/cpr.13198 (PMC9055909; doi:10.1111/cpr.13198)
Supplement: Supplementary file 2 — Figure S2 [file CPR-55-e13198-s008.docx]

**Supplemental Figure 2 Primary antibodies used for this study.**

| **Table 1: Primary antibodies** | | | |
| --- | --- | --- | --- |
| Antibody | Manufacturer | catalog number | dilution |
| ARL13b | Protein Tech | 17711-1-AP | 1:500 |
| Blimp-1/PRDI-BF1 | Cell Signaling Technology | C14A4 | 1:100 |
| Blue (S) Opsin | EMD Millipore | AB5407 | 1:1000 |
| Brn-3 | Santa Cruz Biotechnology | sc-6026 | 1:300 |
| Calretinin (CaR) | Swant | CG1 | 1:400 |
| Chx10 | Santa Cruz Biotechnology | sc-365519 | 1:100 |
| Cone arrestin (ARR3) | Novus Biologicals | NBP1-37003 | 1:500 |
| Cone arrestin (ARR3) | LSBio | LS-C368677-200 | 1:500 |
| CRX | Abnova | H0001406-M02 | 1:1000 |
| C-Terminal Binding Protein-2 | BD Biosciences | 612044 | 1:300 |
| Ki67 | ThermoFisher Scientific | RM-91060-S | 1:200 |
| LHX2 | Santa Cruz Biotechnology | sc-517243 | 1:100 |
| NR2E3/RNR | Abcam | ab172542 | 1:300 |
| NRL | R&D Systems | AF2945 | 1:300 |
| OTX2 | R&D Systems | AF1979 | 1:500 |
| Pax-6 | BioLegend | 901302 | 1:100 |
| Pericentrin (PCN) | Abcam | ab28144 | 1:500 |
| PKCα | Sigma | P4334 | 1:50 |
| Recoverin (RCVN) | EMD Millipore | AB5585 | 1:1000 |
| Red/Green (M/L) Opsin | EMD Millipore | AB5405 | 1:1000 |
| Rhodopsin (RHO) | EMD Millipore | MABN15 | 1:500 |
| Rx | Takara Bio | M229 | 1:400 |
| Sox9 | EMD Millipore | AB5535 | 1:1000 |
| Synuclein γ (SNCG) | Abnova | H00006623-M01A | 1:500 |
| Tubulin β3 (TUBB3) (Tuj1) | BioLegend | 801202 | 1:500 |
