# Supplementary figures and images for: Retinal organoids derived from rhesus macaque iPSCs undergo accelerated differentiation compared to human stem cells
Source: Cell Prolif. 2022 Feb 15;55(4):e13198. doi: 10.1111/cpr.13198 (PMC9055909; doi:10.1111/cpr.13198)

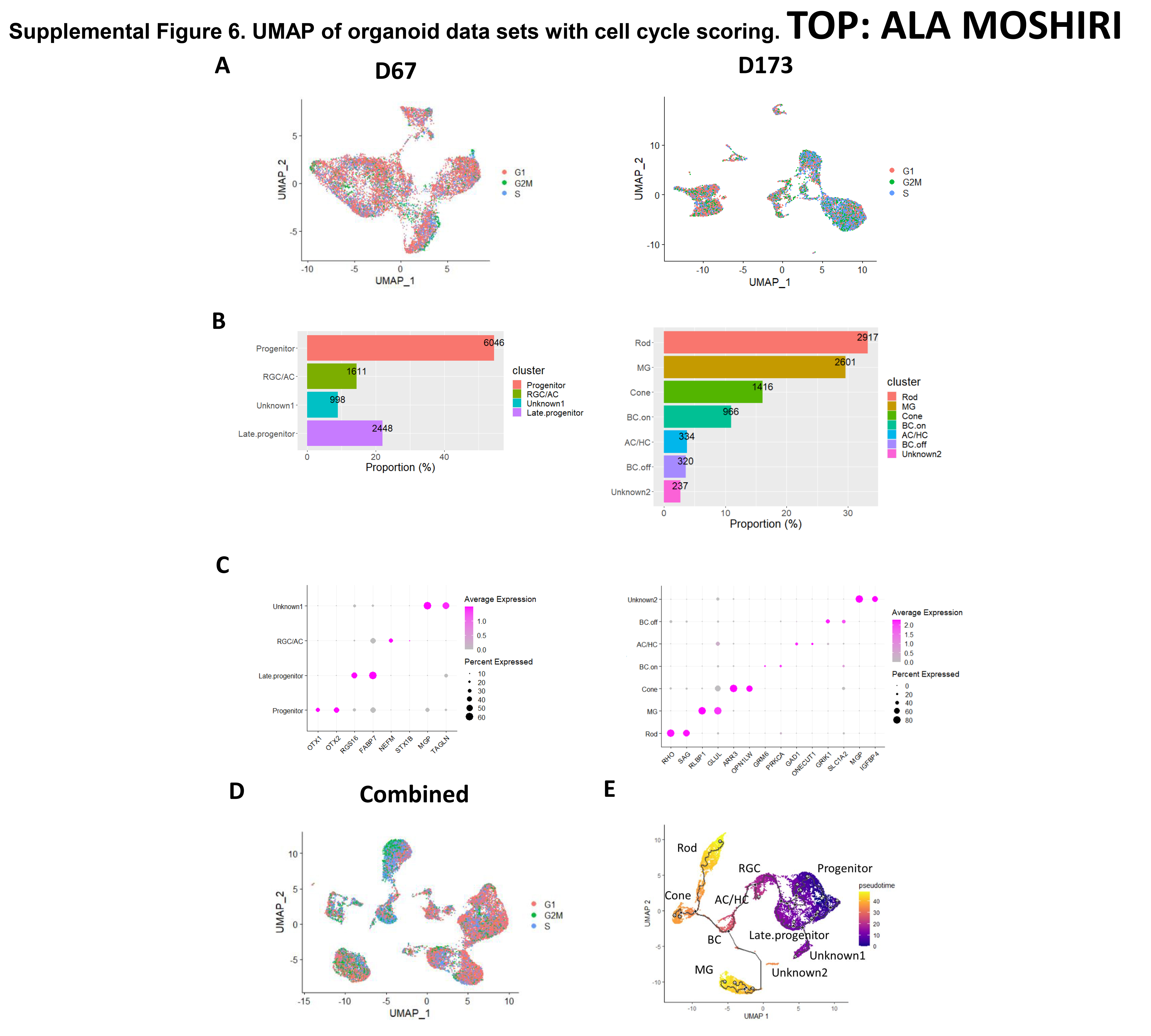

Supplement: Supplementary file 6 — Figure S6 [file CPR-55-e13198-s004.jpg]

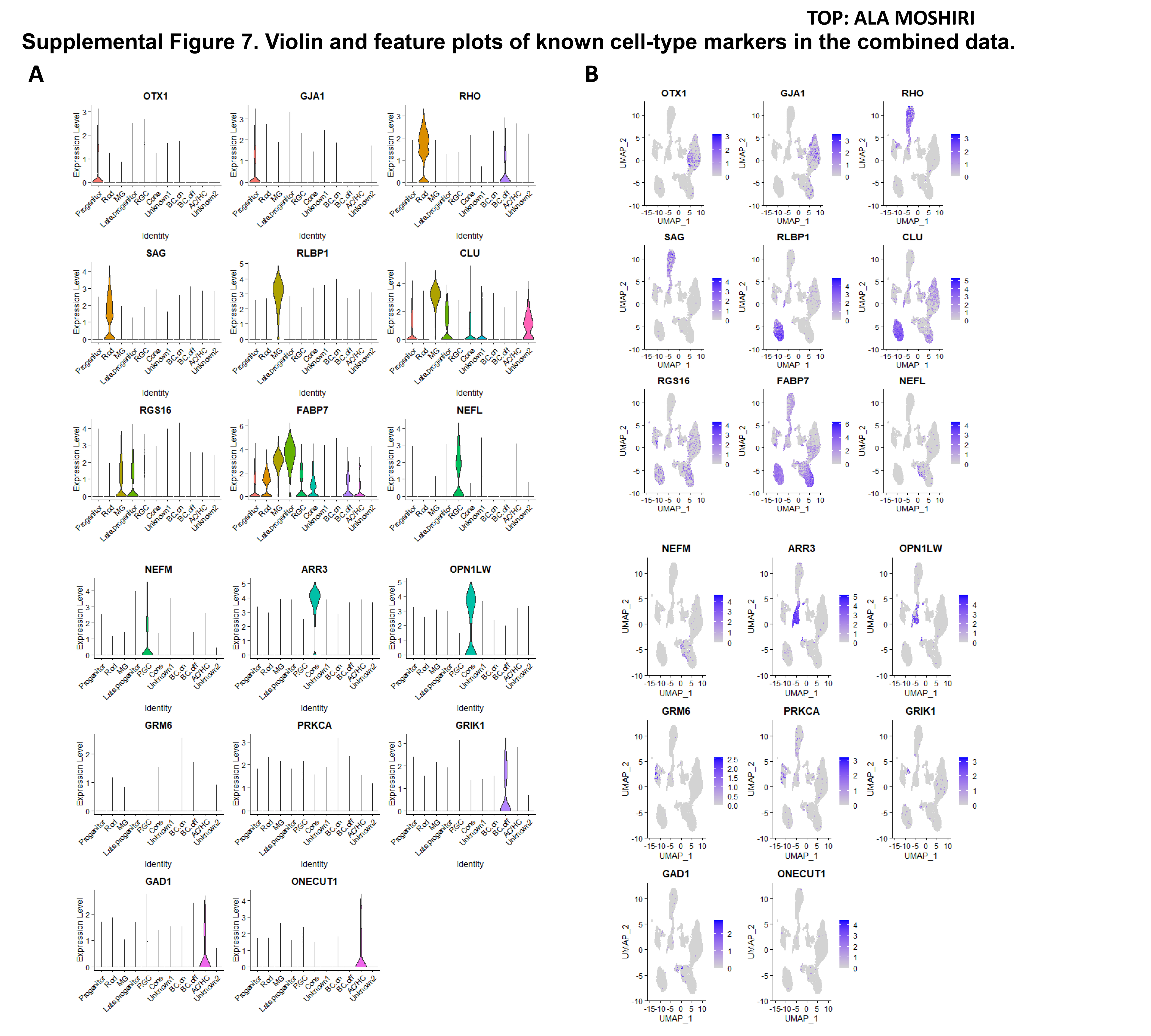

Supplement: Supplementary file 7 — Figure S7 [file CPR-55-e13198-s005.jpg]
